# Leveraging Generative Design and Point Cloud Data to Improve Conformance to Passing Lane Layout

**DOI:** 10.3390/s24020318

**Published:** 2024-01-05

**Authors:** Faeze Momeni Rad, Christoph Sydora, Karim El-Basyouny

**Affiliations:** 1 Department of Civil & Environmental Engineering, University of Alberta, Edmonton, AB T6G 1H9, Canada; basyouny@ualberta.ca; 2 Department of Computing Science, University of Alberta, Edmonton, AB T6G 2E8, Canada; csydora@ualberta.ca

**Keywords:** generative design, BIM, passing lane

## Abstract

Inadequate highway design is a leading cause of traffic accidents, underscoring the importance of adhering to guidelines and regulations for highway design. These standards exist to safeguard road users by addressing crucial factors, like road geometry, signage, and lane markings. Thus, emphasis is placed on computational methods that can optimize towards higher levels of safety, capacity, efficiency, and sustainability in highway designs. Building Information Modeling (BIM) enhances this process by creating a digital model with physical and operational attributes. In this study, a user-friendly, logic-based language is utilized to encode rules for designing highway passing lanes by which designs are automatically evaluated and generated in the BIM-kit software toolkit. This approach is applied to 16 real-world passing lanes in Alberta, showcasing its utility in transportation. The analysis reveals significant enhancements, with rule compliance increasing from 61.82% to 91.31% after employing generative design techniques. These findings underscore the significance of generative design in transportation, offering engineers an efficient tool to create innovative, compliant solutions for highway projects.

## 1. Introduction

The Safe System Approach aims for zero fatalities on transportation networks, acknowledging human error and emphasizing engineering, education, enforcement, evaluation, and engagement (the five Es) for road safety [1]. Engineering involves designing and operating the transportation system, while education focuses on educating road users about safe behaviors. Enforcement ensures compliance with road rules, and evaluation assesses the approach’s effectiveness. Engagement involves collaborating with stakeholders to create support for the Safe System Approach and encourage active participation [2]. Road infrastructure design and engineering are crucial elements in reducing road collisions.

Inadequate highway design contributes significantly to collisions, with road geometry, signage, and lane markings playing crucial roles. They facilitate communication between traffic engineers responsible for designing and regulating traffic flow on roadways and road users, who rely on these signs to navigate the roads safely and efficiently [3]. They offer significant and relevant details regarding the circumstances and restrictions regarding the roadway. Accuracy in designing, maintaining, and evaluating these elements is essential for safe navigation and Autonomous Vehicle guidance. Due to the vast scale of contemporary transportation networks, conventional manual methods for designing signs are inefficient, time-consuming, labour-intensive, and prone to low accuracy. Additionally, such methods can cause traffic disruptions and are economically unfeasible. To improve efficiency, many transportation departments are focusing on automating the sign-designing process.

Finding the best and optimal solution for a specific set of design requirements and constraints involves examining various design choices. Generative design is a process that consists of using algorithms and computational methods to generate and evaluate design solutions. This process encompasses a broad spectrum of techniques and methods utilized in the Computer Aided Design (CAD) domain [4]. The aim of generative highway design is to search for the most optimal solution that meets specific criteria, such as safety, capacity, efficiency, and sustainability. The process typically involves defining the problem, creating a generative design framework, generating design options, evaluating and refining them, and implementing the selected design.

BIM, short for Building Information Modeling, refers to a process that entails developing a structure’s digital model comprising physical and operational attributes [5]. BIM can improve safety in highway design and construction projects in several ways. It can assist with the analysis and simulation of the highway design, enabling engineers to assess multiple scenarios and determine the most efficient and cost-effective solutions. It also allows for creating a comprehensive and precise model that includes various elements, enhancing communication and collaboration between professionals working on the project, such as civil engineers, surveyors, and architects. It enables the creation of 3D models of the highway, which can spark engineers’ creativity and facilitate design visualisation [4].

The BIM-kit [6] is a BIM-based research toolkit for reasoning about BIM models. The toolkit provides a number of Application Programmable Interfaces (APIs) and RESTful web services targeted towards the automation of design review and evaluation, also known as model-checking. Included in the toolkit are generative design services that create design alternatives under provided geometric constraints or rules. The rules for generative design in the BIM-kit are represented in a Domain Specific Language (DSL), or rule language. This rule language simplifies the rule encoding process by using terms more relevant to the BIM model domain [4].

The primary reference for implementing generative design techniques in our research is the *Manual on Uniform Traffic Control Devices* (MUTCD) [7] and the *Highway Geometric Design Guide*, specifically Chapter B, which is published by the government of Alberta [8]. MUTCD is a set of guidelines and standards developed by the Federal Highway Administration (FHWA) to ensure uniformity and consistency in the design, application, and placement of traffic control devices, such as signs, signals, and pavement markings on public roads. Alberta’s Highway Geometric Design Guidelines, on the other hand, offer specific recommendations for road geometric design within the province. Compliance with these guidelines is essential for promoting standardized and safe road infrastructure, benefiting both drivers and pedestrians alike.

The primary objective of this paper is to analyze the design of existing passing lanes on highways located in the province of Alberta. This analysis aims to determine the compatibility of the current passing lanes with the design guidelines. Once the compliance checking step has been completed, the next stage involves proposing generative designs that conform to the relevant code requirements. The designs will be presented optimally, meeting the required standards and specifications. In summary, the objectives of the project can be outlined as follows:

1. Extract relevant highway features and characteristics from point cloud data.
2. Assess the efficacy of current passing lane designs using BIM-kit technology and its rule DSL-based model-checking process.
3. Utilize the BIM-kit’s generative design API to increase compliance with current guidelines and enhance the infrastructure’s design within the existing framework.
4. Augment our understanding of the existing highway conditions and identification of prominent deficiencies in signage placement and accuracy for passing lanes.

## 2. Literature Review

### 2.1. Application of LiDAR in Transportation and Highway Engineering

Light Detection and Ranging (LiDAR) is an optical remote sensing technology that collects data about the surrounding environment using near-infrared light rays. The laser scanners emit beams toward the objects in their vicinity, and by analyzing the characteristics of the reflected beams, they compute the distance to the point at which each beam was reflected [9].

Applying LiDAR data in transportation engineering has garnered considerable interest among researchers and practitioners, leading to a substantial body of literature. Many studies have leveraged LiDAR data to tackle a broad range of challenges in the transportation engineering field. These challenges include assessing road conditions [10,11,12,13,14], monitoring traffic [15,16,17], aiding autonomous vehicle navigation [18], detecting pedestrians [19,20,21,22], facilitating transportation planning by mapping transportation network infrastructures [3,23,24], and other related applications.

LiDAR data plays a crucial role in the development and operation of autonomous vehicles (AVs), serving as a vital component that provides essential information for perception, mapping, localization, and safety. By utilizing LiDAR data to map transportation network infrastructures, transportation planners can gain significant insights and information that can aid in making informed decisions and developing transportation plans that are both effective and efficient, while also being sustainable. LiDAR technology can extract several types of information from paved roadways. These include features like traffic sign information, road markings, and other design elements, such as guardrails, vertical profiles, and light poles. Additionally, LiDAR data can be utilized to obtain roadside clearance parameters, critical for ensuring roadway safety.

A literature review was conducted by Gargoum et al. (2019) that explored the various applications of LiDAR data in transportation engineering [9]. Their evaluation has revealed that most of the past studies in this domain have focused on utilizing LiDAR datasets for inventorying and mapping on-road and roadside features. However, there has not been enough emphasis given to exploring the potential of LiDAR technology to enhance the efficiency of extracting and assessing the geometric design elements of roads. Utilizing innovative techniques and methods to exploit LiDAR data effectively can significantly improve the accuracy and efficiency of road design and maintenance processes. Such advancements could have profound implications for transportation infrastructure, leading to safer and more reliable road networks and ultimately improving mobility and quality of life for individuals and communities.

In summary, the application of LiDAR technology in transportation engineering has tremendous potential to transform the field, and the use of LiDAR technology in transportation engineering has gained significant attention. However, there is a lack of exploration of LiDAR’s potential to improve the efficiency of extracting and evaluating the geometric design elements of roads. Using innovative techniques and methods to exploit LiDAR data effectively, we can significantly enhance the accuracy and efficiency of road design and maintenance processes. These improvements can dramatically impact transportation infrastructure, resulting in safer and more dependable road networks and ultimately benefiting individuals’ and communities’ mobility and quality of life.

### 2.2. Highway Design Assessments

Assessing the suitability of current roads for autonomous vehicles is a necessary step in ensuring that our transportation infrastructure is adequately prepared for the future of transportation [25]. The requirements of autonomous vehicles are distinct from those of conventional vehicles, and the existing road infrastructure may not be adequately equipped to address them. For instance, autonomous vehicles mandate precise and distinct road markings, excellent visibility, and reliable communication infrastructure. The process of reassessing current roads for autonomous driving compatibility includes thoroughly evaluating the road infrastructure and implementing necessary improvements to meet the demands of autonomous vehicles. To improve the reliability and preparedness of Connected and Autonomous Vehicle (CAV) systems, professionals have recommended conducting an evaluation of the current road geometry to identify areas with unfavorable conditions for CAVs and deploying Vehicle to Infrastructure (V2I) communication, which is essential in providing CAVs with necessary information related to the road at critical locations [26].

There are numerous ways to evaluate the design of highways, each with its unique advantages and limitations. Current road maintenance and assessment methods rely heavily on manual surveying techniques and field observations, requiring significant labor and specialized training from transportation agencies. This approach can be time-consuming and costly and may not always provide the most precise results [27]. The predominant method for evaluating highway geometric design is comparing it to established design standards. These standards, usually created and set by national or state transportation agencies based on research, analysis, and best practices, help engineers and designers ensure that the roadway design meets the minimum safety and operational requirements. The characteristics and geometry of the physical features of a road are determined by a variety of factors, including the placement of traffic signs, the presence or absence of shoulders on the sides of the road, the lane width, the sight distance, the location of bus stops, bike lanes, and pedestrian crossing facilities, and the physical separation of the two directions of the road segment by a median barrier. In addition, the horizontal alignment and its associated geometric attributes, such as curvature, slope, and superelevation, are also critical factors that contribute to the overall safety and efficiency of the roadway [28]. By comparing the roadway design to design standards, adjustments can be made to improve the design as needed.

Safety performance functions and simulation modelling are alternative methods utilized for assessing highway geometric design, in addition to comparing the design to established design standards. Safety performance functions use statistical models that correlate the frequency and severity of collisions to different roadway characteristics, such as geometric design features [29,30,31]. These functions help predict the safety performance of various design alternatives and detect hazardous areas needing further safety enhancements. Simulation modelling, on the other hand, is the process of creating a digital representation of a roadway and analyzing the movement of traffic in different situations. This is performed using specialized computer software like VISSIM, a highly useful tool for traffic engineers and transport planners. It helps evaluate highway design and predict traffic flow under different conditions [32]. It should be noted that safety performance functions and simulation modelling are primarily designed to assess the safety of highway geometric design, such as pavement characteristics, roadway alignment, and cross-section design. They do not specifically address traffic sign placement or other elements of highway design.

Consequently, there is a growing demand for exploring alternative approaches utilizing emerging technologies, including machine learning, artificial intelligence, and remote sensing technologies, such as LiDAR [33], to improve road maintenance and assessment efficiency and accuracy. In a recent study, Gouda et al. (2021) introduced an innovative methodology for assessing the sight distance of highways in diverse autonomous vehicle driving scenarios. The approach was based on the utilization of typical commercial vehicle sensor set designs and specifications, providing a novel perspective on the subject matter [18]. The importance of considering all relevant geometric design features in highway assessment cannot be overemphasized. The aforementioned paper appears to have focused exclusively on two critical aspects of highway design: stopping sight distance and speed limit. However, it is crucial to consider other design features as well, given that they also play a vital role in determining the overall safety and efficiency of highways. Consequently, to provide a more comprehensive evaluation of the design features of highways, a framework will be developed within the scope of this paper. Taking a more comprehensive approach to the assessment of highway design features makes it possible to identify potential issues early on and implement appropriate mitigation strategies to ensure that highways remain safe and effective for all users.

### 2.3. Highway Design Methods

Highway design methods are a set of techniques and approaches that transportation planners and engineers employ to create efficient and safe roadways. Designing complex items necessitates thorough exploration of multiple options and possibilities by designers and engineers, who must evaluate various alternatives before ultimately arriving at a final decision [34]. Given the complex nature of highway design and the numerous potential solutions, manual design may not be sufficient to achieve the optimal solution, due to the complexity of the task and the multiple variables that need to be considered [35].

Genetic algorithms (GAs) have emerged as a popular tool in engineering design, finding application in diverse fields, such as mechanical engineering, electrical engineering, aerospace engineering, architecture, civil engineering, and beyond [36]. The flexibility and versatility of GAs make them a valuable resource for designers seeking to optimize complex design problems across a range of domains. Rodriguez-Roman (2018) proposed an optimization model that addressed the joint selection and design of highway safety and travel time improvement projects. The model incorporated a GA that utilized surrogate models to accelerate the discovery of optimal solutions. By leveraging this approach, designers were able to efficiently identify high-quality solutions for the optimization model, resulting in improved highway safety and travel time [37].

In addition, combining Geographic Information System (GIS) and GAs has proven to be a powerful and effective method for tackling complex engineering challenges, especially in transportation. Several research studies have explored the utilization of the integrated approach of GIS and GA for optimizing highway alignments [35,38,39]. Jong aimed to construct a model employing a GIS database and GAs to optimize smooth horizontal alignments that adhere to AASHTO’s minimum-radius constraints. His findings indicated that the proposed model could generate alignments that met highway design standards while also optimizing complex cost functions, including user costs, which had previously been overlooked in many existing models [35].

As implemented in modern CAD platforms, generative design allows architects and engineers to comprehensively investigate many potential design options [34]. It has become a valuable tool in different fields as it allows for exploration of a vast array of design options. The assertion of Shea et al. (2005) that generative design systems aim to create new design processes that generate novel and efficient designs, which are feasible to construct using current computing and manufacturing technologies, accurately represents the current purpose of generative design [40]. To the author’s knowledge, previous attempts to design highways have not given enough attention to generative design as a possible option. This method uses algorithms and computer software to generate multiple design options and select the optimal one based on predefined criteria. Therefore, to bridge this gap, the present study seeks to employ this design approach in highway design.

## 3. Data Description

This paper describes a study that utilized mobile LiDAR technology to collect data from several highways in Alberta, Canada. The Alberta Transportation Agency collected the data using a laser scanner mounted on a moving vehicle that captured information as it travelled along the designated routes. The laser scanner utilized for this study was the REIGL VMX 450, known for its ability to generate highly detailed and accurate 360° LiDAR point clouds. In the case of the RIEGL VMX 450 LiDAR system utilized in this study, it exhibits a documented inaccuracy of 8 mm [41].

The study involved gathering data while traffic was moving, usually at speeds that reached a maximum of 100 km/h, without causing any disruptions to the traffic flow. Provincial surveys were conducted at 90 km/h, resulting in LiDAR point densities ranging from 150 to 1000 points/m^2^ on the pavement surface. The information collected for a specific highway was stored in separate LAS files, each representing a 3D model of a 4 km (≈2.49 mi) section of the road. These files are large, approximately 500 MB, which suggests that the LiDAR model is highly detailed, with vast information on millions of individual data points.

Mobile LiDAR systems can capture data from numerous angles, and this is an advantage that distinguishes them from other LiDAR systems. The various viewpoints allow the system to collect a large number of points, resulting in a higher point density that accurately captures the physical features of the environment. A notable advantage of having a higher point density due to the varied angles from which Mobile LiDAR systems collect data is the improved visibility of vertical elements, including buildings, signs, and trees [27]. These features’ detailed and accurate portrayal provides valuable information about their dimensions and distances, enabling better comprehension and interpretation.

**Figure 1:**
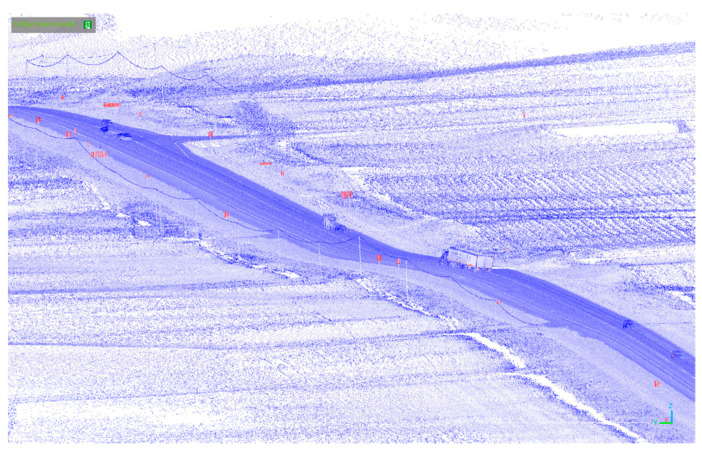
Virtual image of the LiDAR point cloud sample for a passing lane.

The Mobile LiDAR data gathered on each point comprises various parameters, such as *x*, *y*, and *z* coordinates, intensity measurements and scanning angle information. The LiDAR point cloud for one of the passing lane segments is presented as a virtual image in Figure 1. The highlighted red areas in the figure represent features with higher intensity in comparison to others, with traffic signs predominantly standing out within these regions.

**Figure 2:**
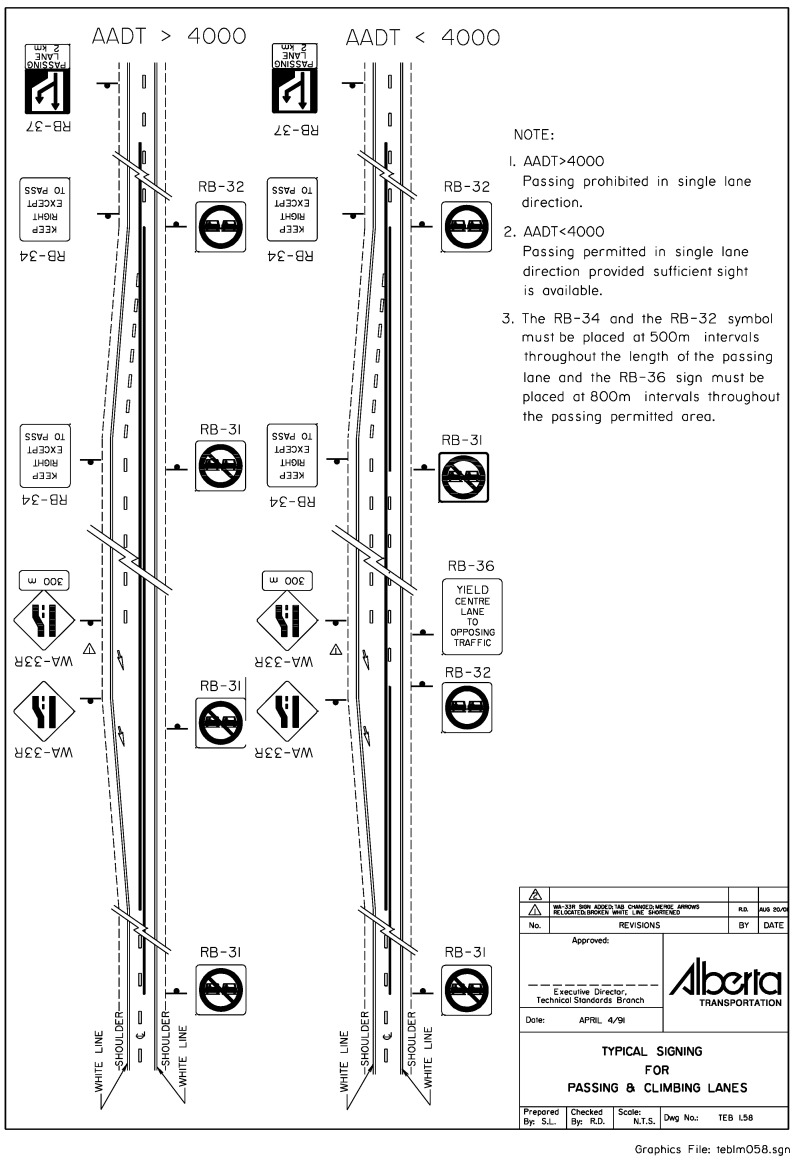
Signing for Passing and Climbing Lanes.

A passing lane, also referred to as an overtaking lane or a fast lane, is a designated portion of a roadway or highway that facilitates smoother traffic flow and enhances safety by providing an extra lane for faster vehicles to pass slower ones without disrupting the overall traffic movement. Figure 2 displays the Passing and Climbing Lanes signage sourced from Alberta Transportation [8].

Table 1 presents the traffic signs utilized in the study, along with their corresponding descriptions and shapes.

The study utilized LiDAR data specifically acquired for Highway 3 and Highway 9 in the province of Alberta. The objective was to assess the effectiveness of a method on various passing lanes, each exhibiting distinct characteristics, and across different highways.

**Figure 3:**
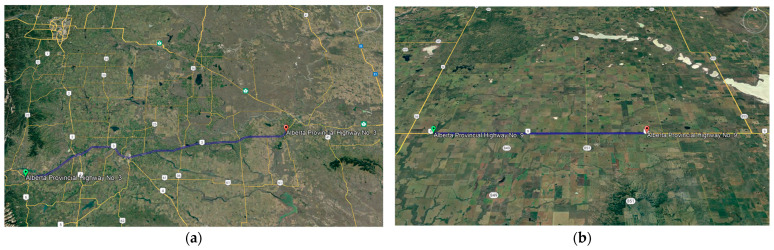
Highway Locations on Google Earth: (**a**) Alberta Provincial Highway No. 3; (**b**) Alberta Provincial Highway No. 9.

Alberta Provincial Highway No. 3, also known as the Crowsnest Highway, is a provincial road in Alberta, Canada, spanning approximately 324 km (201 miles) as it crosses the province’s southern region. Alberta Provincial Highway No. 9, located in the south-central region of Alberta, Canada, serves as a crucial connection between Calgary and Saskatoon, Saskatchewan, via Drumheller, in partnership with Saskatchewan Highway 7. These sections, which feature passing lanes, are part of a two-way, two-lane highway. For our analysis, we focused on 4 km segments of these highways, each averaging approximately 1.98 km in length. Figure 3 displays the positioning of these highways.

## 4. Research Methodology

**Figure 4:**
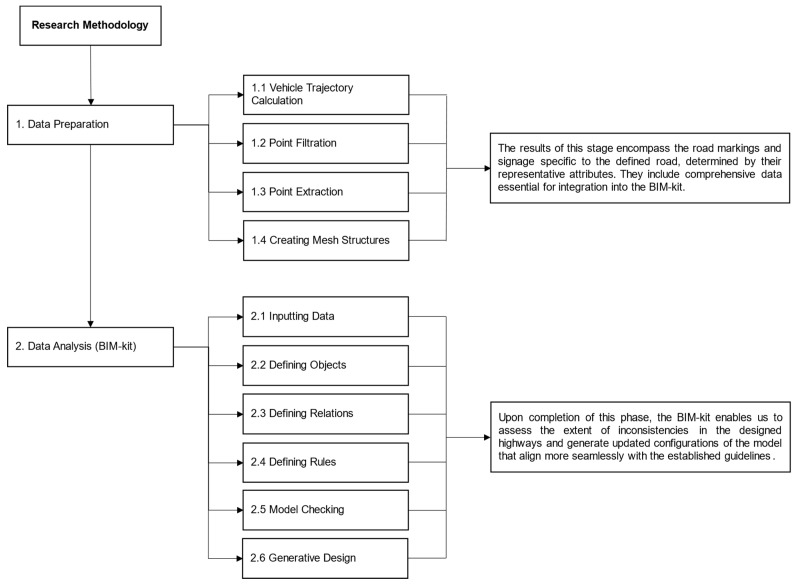
Summary of the Research Framework.

To attain the research objective, the methodology employed is illustrated in Figure 4 and carried out in two primary stages: (i) data preparation and (ii) data analysis, utilizing the BIM-kit. The analysis involved the utilization of highway models to evaluate the degree of inconsistencies in the designed highways, ultimately producing revised configurations of the model that adhere more cohesively to established guidelines.

### 4.1. Vehicle Trajectory Calculation

In this study, the vehicle utilized for the data collection process was equipped with two LiDAR scanners that were symmetrically positioned. The LiDAR scanners played a vital role in deriving the vehicle’s trajectory during the data collection phase. Data points located directly underneath it with a scanning angle of zero were utilized to obtain the trajectory of each scanner. The overall trajectory of the vehicle was obtained by averaging the trajectories of the two scanners while using GPS time as a reference. The equation for the calculation of the line is provided below:

(1) $$LinePoints=\left\{\frac{S_{1_{i}}+S_{2_{i}}}{2}|\left(SA\left(S_{1_{i}}\right)=SA\left(S_{2_{j}}\right)\right)=0\wedge \left(T\left(S_{1_{i}}\right)=T\left(S_{2_{j}}\right)\right)\right\}$$

*SA*(*x*) denotes the scan angle of the LiDAR scanner, while *T*(*x*) is the GPS time recorded during data collection. *S*_1_ and *S*_2_ refer to the sets of trajectory points for scanner 1 and scanner 2, respectively. To obtain the trajectory of the vehicle, the indices *i* and *j* are used to reference the points in sets *S*_1_ and *S*_2_, respectively. The equation considers all these factors and produces a line accurately representing the vehicle’s overall trajectory during data collection.

### 4.2. Point Filtration

Point cloud information, collected in the LAS (laser) format, requires conversion to ASCII text format for easier data filtering. The conversion of point cloud data from LAS (laser) format to ASCII text format is essential due to the superior human readability and editability offered by ASCII text. This facilitates a more straightforward and comprehensible inspection of the data, a critical aspect during the filtering phase. Unlike the binary LAS format, ASCII text ensures a format that is easily readable by humans. Additionally, the adoption of ASCII text format ensures enhanced interoperability across diverse software and platforms. This is especially pertinent, as numerous data processing and analysis tools are designed to seamlessly support ASCII text formats, including compatibility with the BIM-kit. As a result, the conversion to ASCII text not only improves accessibility, flexibility, and compatibility during the filtering process, but also contributes to a more efficient and user-friendly approach to data analysis.

A three-step filtering process detects lane markings and traffic signs in a LiDAR point cloud. The first filter identifies the points close to the expected vehicle trajectory, which is determined by analyzing the scanning angle of the LiDAR data within a certain buffer zone. By extracting the points located near the trajectory points, which cover the limits of travel lanes and roadside, the algorithm can optimize the number of points processed, reducing the computational load. According to the prescribed guidelines, the shoulder adjacent to a passing lane should be no less than 1.5 m wide or equal to the standard shoulder-width designated for that particular highway design, whichever is smaller. Hence, this paper assumes a maximum shoulder width of 2 m to account for potential design errors. To keep the point clouds covering the centerline lane markings and signs on two-lane two-way highways with lane widths ranging from 3.5 m (≈11.48 ft) to 5.0 m (≈16.40 ft), the filter is set to keep the points located at a distance between 4.5 m (≈14.76 ft) and 12.0 m (≈39.37 ft) from the vehicle trajectory.

The following filter to extract lane markings eliminated those with high z-gradient values, which typically correspond to roadside features and buildings, and only preserved the road boundaries for further analysis. It is important to note that, in contrast, low z-gradient values were removed while extracting traffic signs. z-gradient is determined using the subsequent method:

(2) $$\vec{\nabla }f_{p}=\frac{1}{k}\sum_{i=1}^{k}\frac{\vec{PP_{i}}}{\left\|\vec{PP_{i}}\right\|}\left(f\left(P_{i}\right)-f\left(P\right)\right)$$

The *z*-value of point *x* is denoted by *f*(*x*), where P represents the point for which the gradient is calculated. *P_i_* refers to the points in a group of *k* neighbouring points evenly sampled within a radius around the given point.

The third filtering step involves using an intensity-based filter that takes advantage of the information provided by the LiDAR data. In particular, the LiDAR data contains information about each point’s position, elevation, and intensity in the point cloud. A heuristic approach was adopted to determine the optimal intensity threshold for filtering. The threshold is gradually reduced until lane markings and most traffic signs are detected, even if other objects are retained. Highly reflective objects, such as traffic signs and lane markings, have high-intensity readings compared to other points. Traffic signs must be painted with retroreflective material to meet the visibility requirements of the MUTCD under poor lighting conditions. As a result, by filtering LiDAR points by intensity, high-reflectivity points, such as traffic signs and lane markings, can be preserved. Thus, the intensity filter is applied to isolate traffic signs and lane markings from other objects in the point cloud data. Since the traffic signs and lane markings are the highly reflective objects among the previously filtered group of points, the filter successfully separates the traffic signs and lane markings from other points. Then, the process of removing outlying points was carried out using statistical outlier removal (SOR).

### 4.3. Point Extraction

The next step involves utilizing the refined cloud to establish passing lane zones and extract their characteristics. Within the filtered cloud, distinct signs and lane markings are detected by clustering adjacent points into groups using Density-Based Spatial Clustering of Applications with Noise (DBSCAN). This technique groups points by proximity and number of hits, where the hit count refers to the number of points in a cluster. Proximity limits the distance between points that can be deemed as part of the same cluster, excluding small groups or points that are far apart. Suitable values for proximity and hit count were selected to yield optimal outcomes.

Typical lane markings include both dashed lines, each 3 m (≈9.84 ft) in length and spaced 6 m (≈19.69 ft) apart, as well as solid lines. To define clusters of dashed lines, a threshold of 3 m (≈9.84 ft) is used, based on the geometry of the markings and the density of the LiDAR data (300 points/m^2^). Clusters containing between 10 and 100 points and a length of up to 3 m (≈9.84 ft) are identified as “dashed” marking lines, while clusters with over 100 points and a length greater than 3 m (≈9.84 ft) are classified as “solid” marking lines. Using the standard lengths of “dashed” and “solid” lane markings, the successful extraction of lane markings and identification of passing lane zones was possible for all analyzed highway segments using these criteria. These results will be used for model verification and generative design for each road segment.

A minimum hit count threshold of 17 was used to identify traffic sign clusters, meaning that any group with fewer than 17 points was not considered as a cluster. Additionally, a proximity parameter threshold of 1.0 m was used, such that if the distance between adjacent points in a group was greater than 1 m, those points were not included in the same cluster. These specific thresholds were chosen because they were the most effective in minimizing the identification of other high-intensity objects, such as road markings and license plates, and in identifying only traffic sign clusters. The thresholds selected underwent empirical validation through a process of iterative testing and experimentation. Various values for proximity and hit count were systematically tested on sample datasets to assess their impact on identifying lane markings and traffic signs. Next, ground truth data on traffic signs along highway segments were gathered manually from Google Street View. Images were visually verified, and the name of each sign, along with the number of signs on each segment, was documented.

### 4.4. Transforming Point Data into 3D (or 2D) Mesh Structures

Upon completing the extraction of passing lane characteristics, we proceed to the subsequent stage of our analysis. It is important to note that, although features such as intensity were beneficial in the previous step, we now exclusively focus on the *XYZ* columns, as they contain the most relevant information. These columns are then arranged based on their direction vector along the *x*-axis of the point cloud data.

When analyzing lane markings, the typical recommendation is to arrange them according to the direction vector of all points, using Principal Component Analysis (PCA) to identify the road direction. Nevertheless, it has been noticed that the majority of these markings tend to align with the *x*-axis direction. This observation allows us to temporarily justify the assumption of sorting them based on the *x*-axis direction vector. When roads were not aligned with the *x*-direction, a rotation was applied to all the points within the passing lane, effectively aligning them with the *x*-direction. This adjustment proved highly advantageous, greatly simplifying the subsequent stages of the analysis. For the sign files, it is advisable to utilize the road direction vector and consider how the forward and backward directions of the signs are determined.

In the road representation context, addressing the abundance of points present along the road network is essential. Given the extensive length of roads, utilizing bounding shapes becomes imperative to prevent the formation of an excessively large encompassing region. To achieve this, the points are effectively partitioned into smaller groups, each containing a specified number of points, denoted as N. Through experimentation, N has been determined to be 1000. In the given context, opting for a grouping size of 1000 points proves suitable, due to its capacity to facilitate efficient data processing, grant control over bounding shapes, sustain an acceptable point density, and its empirical validation as an appropriate parameter for mitigating the challenges associated with the abundance of Lidar points within the road network. As previously noted, it is worth emphasizing that Lidar point densities on the road surface may exhibit variations ranging from 150 to 1000 points per square meter. By electing N as 1000, the assurance is that, on average, each group encapsulates an area equivalent to 1 square meter (calculated as 1000 points divided by 1000 points per square meter). This meticulous approach contributes to the preservation of an appropriate point density within each group, thereby enabling the accurate capture of intricate road surface details. It is worth noting that selecting an excessively large value for N would result in a single, cohesive entity, whereas opting for an excessively small value would introduce irregular gaps and numerous shapes with a high triangle count, consequently impeding search efficiency. Consequently, each group of points is treated as an individual entity rather than being considered as mere components of a single comprehensive road structure. This distinction holds significant importance and serves as a foundation for the subsequent discussion on the relationships we introduce, which will be further elaborated.

When it comes to grouping points, there are two effective strategies to consider. The first approach involves creating a bounding box, which encapsulates the points within a rectangular region given the orientation of the box. This method is beneficial for simplifying the representation of the road network. The second approach is to derive the convex hull, which determines the smallest convex polygon enclosing all the points. In both cases, the road segment orientation is described by the direction of the road segment and the upward, *z*-axis. In our implementation opted for a convex hull from a top-down XY perspective, then extruding the shape based on the minimum and maximum *z*-values, the resulting volume captures the three-dimensional characteristics of the road. This ensured that the shape of the road segment was maintained, while keeping a reduced polygon count for the computationally intense geometrical calculations.

In the case of two-dimensional scenarios where a third dimension is irrelevant, employing either the bounding box or the convex hull method is sufficient. Again, we opted for the convex hull, resulting in a polygonal shape that tightly wraps around the points, accounting for their spatial arrangement.

The ear clipping technique is utilized to achieve a triangulated representation of the road shape. This algorithm effectively decomposes the shape into a series of triangles by progressively removing ear-like regions from the polygon until all remaining vertices are connected. The resulting triangulation facilitates efficient rendering and spatial analysis of the road geometry.

The representation of signs follows a straightforward procedure. To begin with, the sign’s shape can be derived by employing the convex hull method when observing it from a top-down perspective. This allows for accurate capturing of the sign’s boundaries. It is extruded along the *z*-axis to add depth to the sign, resulting in a three-dimensional representation.

Notably, sign objects typically do not necessitate the splitting of points, as the number of points in a sign file tends to remain below 1000. Thus, the complexity associated with point grouping and separation, often encountered in the case of roads, is significantly reduced for signs. This streamlines the data processing pipeline, enabling more efficient handling of sign-related information.

Each sign file is named after its corresponding object to ensure systematic organization. This naming convention facilitates easy retrieval and association of data during subsequent analysis. Additionally, the type “Sign” is assigned to these files, aiding in the classification and categorization of sign objects within the broader context of the research.

### 4.5. Rule Language

The rule language is a Domain Specific Language (DSL) for describing rules as part of the larger BIM-kit model evaluation and reasoning platform [5]. Briefly, the BIM-kit consists of a variety of applications and automation services that aid in the reasoning of BIM models. Included in the BIM-kit toolkit are a model viewer and editor, rule language editors and repository, and rule language-based model checking and generative design services. The rule language is designed to express various types of rules, including geometrical and non-geometrical properties of objects, whether actual or virtual elements, and geometrical relations between pairs of elements. Additionally, the rule language allows for the definition of complex rules that involve logical compositions of the aforementioned basic rules. The design of this rule language aims to provide a flexible and comprehensive framework for describing and analyzing properties and relationships within the BIM-kit system.

Several geometric rules were introduced in the preceding study [4], derived primarily from interior rules. It is important to note that, despite these new introductions, numerous fundamental relationships, such as distance, facing, and above, remain applicable and relevant in the current context. These enduring relationships play a crucial role in the ongoing analysis. Three additional relations were introduced to enhance the rule expressiveness within the system. Firstly, the RoadPathDistance relation was implemented to address the limitations of measuring distances “as the crow flies,” or the standard distance calculations, which typically consider the shortest straight-line distance between two points. Instead, RoadPathDistance accounts for the actual road network by determining the nearest road segment and corresponding point to the start point. This process is repeated iteratively, finding the closest road segment and pointing to the previous point until reaching the road segment nearest to the goal point. By incorporating road paths, the RoadPathDistance relation provides a more accurate distance measure along the road network.

Secondly, the LeftSideOf relation was introduced, which involves calculating the cross product between the vector from the center of an object to a specific point and the object’s forward direction from its center. If the cross-product result is greater than 0, the relation evaluates to true; otherwise, it evaluates to false. This relation helps determine whether a given point lies on the left side of an object regarding its orientation.

Conversely, the RightSideOf relation serves as the opposite of the LeftSideOf relation. It also involves calculating the cross product between the vector from the center of an object to a point and the object’s forward direction. If the cross-product result is greater than 0, the relation evaluates to true; otherwise, it evaluates to false. Table 2 presents the geometric relations employed to enhance rule expressiveness.

Within the BIM-kit’s framework, an inventory of objects has been defined, encompassing various elements. Several new object types were introduced to augment the existing list as part of the expansion process. Notably, the inclusion of road-related information posed a unique challenge, as it was not originally incorporated into the Industry Foundation Classes (IFC) type list that serves as the foundation for BIM-kit’s type list. The new types were categorized under the CivilElement type classification to address this. The newly added object types are as follows:

1. Road
2. Sign
3. Various Road Markings (such as RoadDivergePoint, RoadConvergePoint, and more)

These Road Markings are considered as “virtual objects” in the Rule Language paper, possessing geometry despite their non-physical nature. By incorporating these new types, the BIM-kit’s framework expands its scope to encompass road-related elements, allowing for the formulation of rules that account for these objects’ unique characteristics and interactions. This extension enhances the rule expressiveness of the framework and facilitates a more comprehensive analysis and manipulation of road geometry within the BIM context. Table 3 presents a complete description of each object and its corresponding name utilized for the analysis.

The lack of precise labelling for individual points presents a significant obstacle in accurately determining the exact nature of the road. This challenge is further compounded when dealing with point clouds that contain missing data, leading to irregular and unpredictable shapes. Given these limitations, the most practical approach is to analyze the road in a three-dimensional context and manually assign markings to the points based on a combination of road layout knowledge and visual inspection.

To facilitate this process, the BIM-kit model editor plays a crucial role, offering the capability to incorporate dummy objects into the object catalogue. This functionality enables users to manually add the necessary markings, thereby enhancing the interpretation of the road shape and contributing to a more comprehensive understanding of the entire road network.

### 4.6. Model Checking

The rule-based model compliance checking process consists of four essential stages [4]: rule interpretation, model preparation, rule execution, and reporting the checking results. In the first stage, natural language design constraints are transformed into a rule language that computers can understand, enabling the automation of rule interpretation. This step is crucial for ensuring the rule language can be processed effectively.

This study’s interpretation phase involves manually interpreting the rules’ logic into the appropriate rule language using a rule editor interface. Once all the rules have been created, they are exported to a file that the model check application can read. The rules utilized in this study have been extracted from the *Highway Geometric Design Guide of Transportation Alberta* and can be found in Table 4.

A systematic approach is employed for writing the rules to accommodate the absence of road splitting based on direction. The rules are developed and evaluated separately for each direction of the road. Initially, the focus is on checking the road signs positioned on the right side of the road. Once this evaluation is complete, the direction of the road is flipped, and the rules are applied to assess the other side of the road. This sequential methodology acknowledges the current road configuration and ensures that rule considerations align with the specific direction of the road. Although the issue of road splitting remains unaddressed due to its inherent complexity, it is recognized that modifying the rules and introducing additional properties may be necessary if road splitting were to be implemented.

In the provided table, three specific rules were designated as error-related rules (or Hard Constraint), which held utmost importance and had to be fully satisfied without compromise. These rules specifically centered around the positioning of traffic signs, with the criteria encompassing situations where (a) signs were situated too close or too far from the road, (b) signs were positioned above the road, and (c) maintaining a minimum distance of 1 m between two signs was required to avoid duplications.

Notably, these error-related rules were non-negotiable and required strict adherence in the context of generative design. These rules were deemed critical, as they directly influenced the effectiveness and safety of the passing lane. Any deviation or failure to comply with these rules would be considered a substantial error and necessitate corrective action.

Conversely, the remaining rules were classified as warning-related rules (Soft Constraint). These warning-related rules encompassed a broader range of important criteria to consider but carried a lesser degree of severity regarding their impact on the passing lane’s functionality and safety. Although these rules demanded attention and compliance, they were comparatively less critical than error-related ones.

The second stage, model preparation, involves dealing with the information contained within the model. Sometimes, this information may be disorganized or not explicitly defined, resulting in slow and laborious checking procedures. To address this, the second stage focuses on organizing and summarizing the model’s information, making it easier and more efficient to perform the compliance checks. It has been observed that the efficiency of the rule checks significantly improves when the road and signs are represented in a 2D format. This involves adjusting the *z*-value of the points to zero and eliminating the extrusion process. The number of triangles comprising the objects is reduced by transforming the road and signs into a 2D representation, thereby accelerating the rule evaluation process. This simplification technique potentially reduces the overall check time by approximately one-third. Consequently, the system benefits from improved computational efficiency, enabling faster and more streamlined rule assessments.

During the rule execution stage, the organized model information is used to evaluate each rule sequentially. The compliance checking process can determine whether each rule is satisfied or violated by applying the rule-based approach based on the available model data.

Finally, the last stage involves providing the user with a comprehensive report of the checking results. This report includes a rule-by-rule evaluation, indicating the compliance assessment of the model. Additionally, it contains information about any faults or issues identified during the checking process, which were instrumental in determining the overall compliance status.

Following these four stages, rule-based model compliance checking can be conducted professionally, ensuring that design constraints are accurately interpreted, model information is effectively organized, rules are executed systematically, and detailed reports are generated to facilitate compliance evaluation.

### 4.7. Generative Design

Enhancing the design of passing lanes can prove to be a demanding endeavor, primarily due to the many design restrictions that must be considered. However, by harnessing the power of computational methods to generate and assess various design alternatives, the time required for this process can be significantly reduced [42]. The generative design process necessitates the inclusion of three key components: a Performance Metric, a Configuration Variation method, and a Decision-Making Response.

In assessing the quality of each stage within the generative design process, the method proposes the adoption of rule-based compliance checking as the performance metric. The previously described model-checking approach is employed, albeit with specific adjustments to the four stages involved. The first alteration occurs during the model preparation stage, adding missing traffic signs. While static model objects do not necessitate property recalculation, the addition or movement of an object requires recalculating all its relationships with other objects. The second alteration occurs during the execution stage. While a binary true or false value suffices for model-checking purposes, it is advantageous in generative design to scale the outcome of the rules. Checks on Boolean and string properties only yield a true or false result, corresponding to values of 1 or 0, respectively. However, a numeric property check can be formulated to produce a result within the range of (0, 1). A value of 1 signifies full compliance with the rules, whereas any value less than 1 indicates varying degrees of rule violation.

In the context of configuration variation, the generative design approach requires three inputs: a Building Information Modeling (BIM) model representing an empty space, functioning as the passing lane layout, a set of objects consisting of traffic signs to be integrated, and a predefined set of design rules. This method utilizes these inputs to generate fresh configurations of the model. The procedure involves identifying suitable objects to include and determining their optimal placement locations within the design. Notably, in this specific case, the objects in focus pertain exclusively to traffic signs. Furthermore, it is noteworthy that, during the generative design process, adjustments to the orientation or location *z*-value are deliberately omitted due to their typically apparent and self-evident nature on-site.

The decision-making process is characterized by a meticulous evaluation of various configuration changes, each undergoing thorough scrutiny. Subsequently, a selection is made based on these evaluations to identify the most suitable configuration for further progression. On the other hand, the generative design approach adopts a greedy strategy, wherein it systematically chooses the configuration with the most favourable evaluation and iteratively continues the process from that chosen point.

Moreover, the process does not merely involve a single round of evaluation; instead, it is an ongoing and iterative procedure. As new information becomes available or the project progresses, the decision-making process may adapt to incorporate new considerations or reevaluate existing choices.

## 5. Results and Discussion

To evaluate the effectiveness of the generative design methodology, experiments were conducted on 16 real-world passing lane scenarios. The approach involved applying model-checking techniques to the available passing lane samples. Initially, the point cloud data was transformed into a 3D mesh representation, creating a model of the existing lanes. By leveraging the features of these lanes and the generated model, the model-check evaluation method from the model-check library was used to calculate scores for each road segment.

The outcomes of the model-checking procedure provided pass or fail results for each utilized rule. These rule outcomes included Boolean values and corresponding references with detailed rule descriptions. Additionally, the results encompassed information about each rule instance and its outcome, ensuring a thorough evaluation and analysis of individual rule performance.

The generative design methodology’s effectiveness and accuracy in generating passing lane designs that deviate from the guidelines were assessed through this evaluation process. The flexibility and adaptability of this methodology in addressing unique requirements in passing lane design were demonstrated by generating designs that differed from existing norms.

In the following section, one of the passing lane scenarios is detailed, both before and after the application of the generative design approach, serving as examples. The results of each design iteration are presented at the end of this section, showcasing the outcomes achieved through the generative design process.

### 5.1. Passing Lane Example

**Figure 5:**
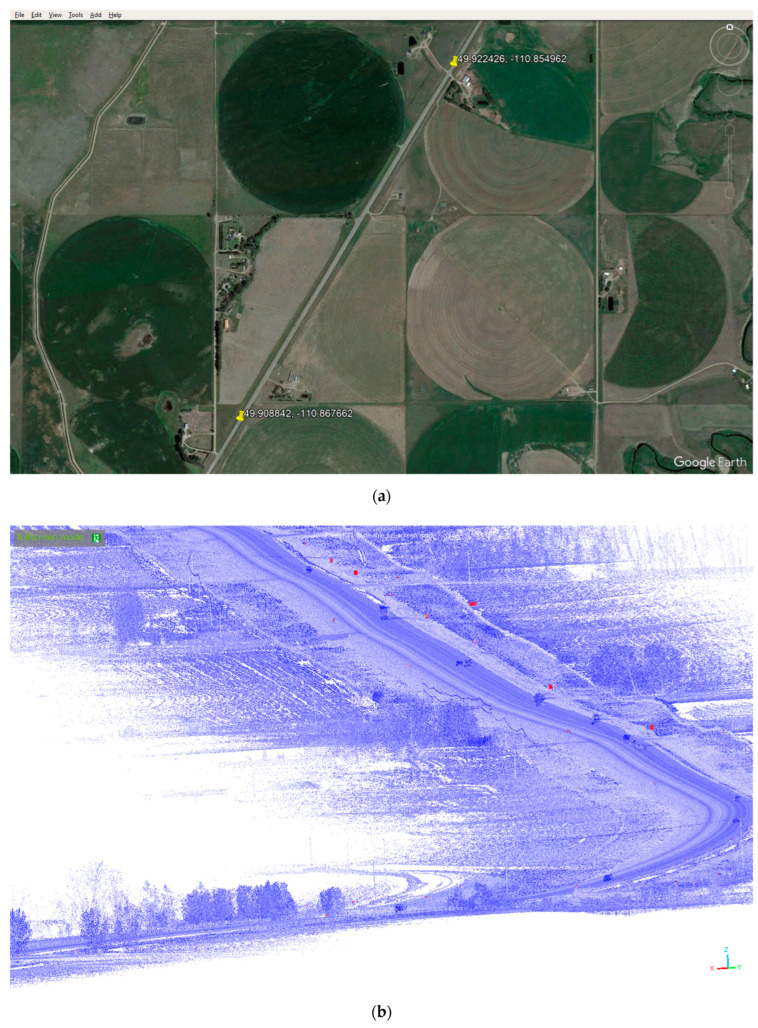

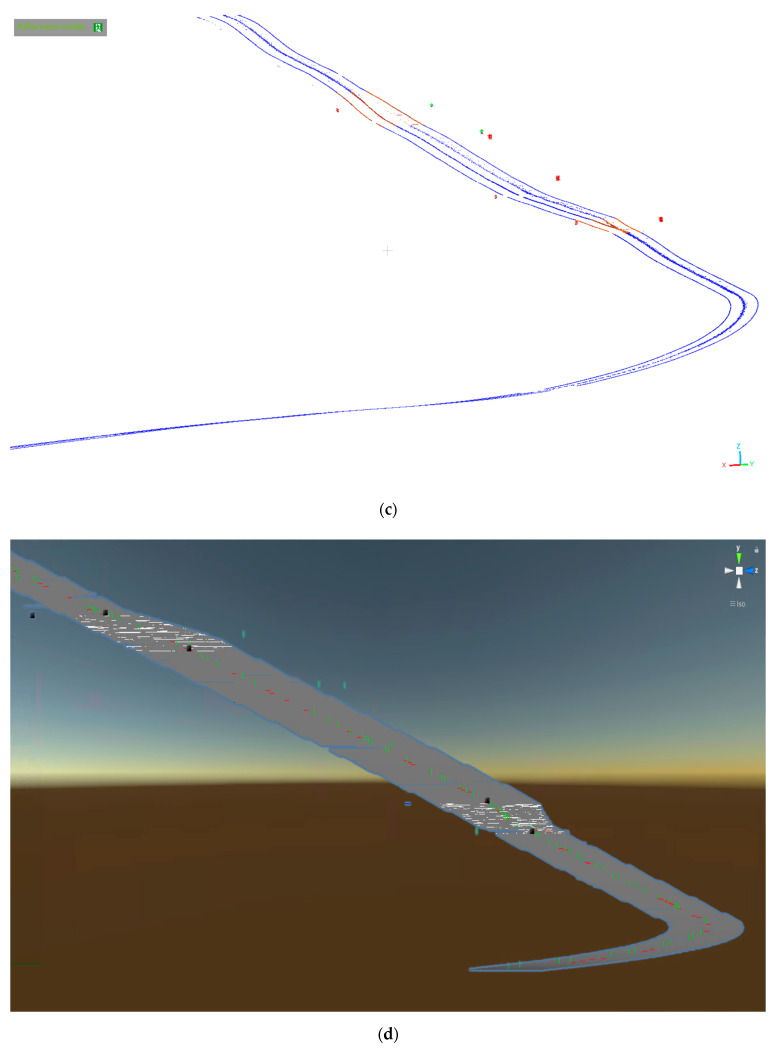
Visual representation of this passing lane: (**a**) satellite image location; (**b**) raw point-cloud; (**c**) filtered point-cloud; (**d**) generative design result.

The passing lane in question was located on Highway 3, more commonly called Crowsnest Highway. Spanning a distance of 1.8 km, the passing lane extends from coordinates 49.922426, −110.854962 to 49.908842, −110.867662, as illustrated in Figure 5a.

The passing lane in question has been assessed for its compatibility with the existing guidelines, yielding a compatibility score of 58.611%. This score indicates that, out of the 18 rules applied to evaluate the passing lane, the total score of these rules amounted to 10.550. It is important to note that 10.550 does not necessarily mean that ten rules passed. The evaluation revealed that, apart from three rules that received a perfect score of 1 and passed, all others scored below 1. For example, the “WA-33R Location 2” rule received a score of 0.40. Breaking this down further, within this set of rules, the score for error-related rules amounted to 2.615 out of 3, suggesting a high level of compliance. Two of these three rules received a score of 1, but the “Sign Road Dist.” rule failed, obtaining a score of 0.61. However, when considering the warning-related rules, the score was 7.935 out of 15, revealing that the total failure score for rule-compliances in this passing lane is 7.065.

Regarding signage, the passing lane exhibits an additional RB-34 sign while lacking one RB-31 sign. To rectify this issue, necessary adjustments were implemented to ensure the inclusion of the correct number of signs and the incorporation of any missing signs into the model. The generative design runtime for this passing lane amounted to 02:41:38, involving a comprehensive check count of 21,840. (The device uses for analysis is equipped with a 12th Gen Intel(R) Core(TM) i9-12900KF processor running at 3.19 GHz, 64.0 GB of installed RAM (63.8 GB usable) and operates on a 64-bit system with no pen or touch input available for its display.)

After implementing the generative design approach, there has been a notable increase in the compatibility score of this passing lane, reaching an impressive 93.196%. This substantial improvement indicates that, out of the 18 rules considered in the evaluation, a significant majority of 16.775 rules were successfully satisfied by this passing lane.

It is worth noting that all three error-related rules were met within this set of rules, demonstrating a flawless adherence to those specific guidelines. Furthermore, when it comes to the warning-related rules, this passing lane exhibited compatibility with 13.775 out of the total of 15, leaving only 1.225 rules that have not been fully met. This achievement highlights the high level of compliance achieved by the passing lane regarding the warning-related criteria, with room for further optimization to fully satisfy all the rules. The scores for each of the rules, both before and after the implementation of generative design, are presented in Table 5.

By undertaking the generative design process and making necessary adjustments, the passing lane achieved notable enhancements in overall score and adherence to guidelines. To provide a visual representation of this passing lane and its associated data, Figure 5 serves as a point of reference. The figure comprises several parts that offer valuable insights into the passing lane’s characteristics. Part a of the figure showcases the precise location of the passing lane. Part b presents the raw point-cloud file corresponding to this passing lane, while part c showcases the extracted features and characteristics specific to this particular passing lane. Finally, part d illustrates the design of the passing lane after applying the generative design process.

### 5.2. Summary of Results

**Figure 6:**
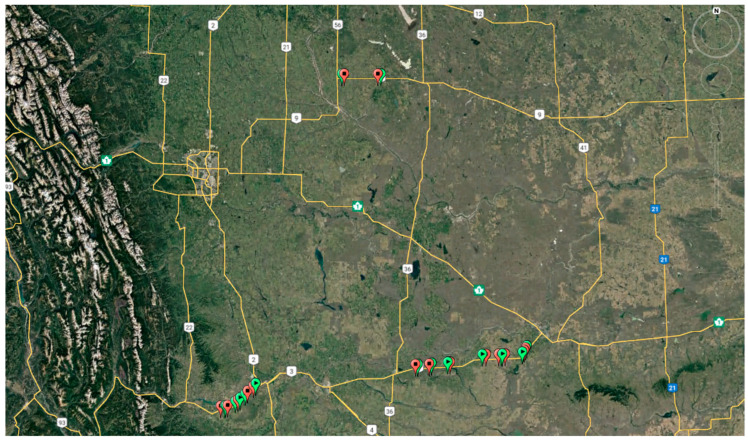
Passing lane locations (green indicates the start of a passing lane, and red designates its end).

A series of experiments were conducted on 16 real-world passing lane scenarios to assess the effectiveness of the generative design methodology. Figure 6 provides a visual representation of these passing lane locations.

The methodology involved applying model-checking techniques to the available passing lane samples, followed by implementing generative design to reduce their level of incompatibility with established guidelines. This evaluation aimed to determine the efficacy and accuracy of the generative design methodology in producing passing lane designs that deviate from the guidelines.

One passing lane scenario was discussed and, detailed information was provided, encompassing the specific highway location, precise start and end coordinates, passing lane length, initial real-world score, score after applying generative design, the runtime of the generative design process, and the number of checks performed. Visual representations were also presented, including the passing lane’s location, the raw point-cloud data, the filtered point-cloud, and the design generated through generative design.

In this study, approximately 31.6 km, which featured a total of 16 passing lanes, underwent comprehensive examination. It was observed that, among the various passing lane signs, the RB-31 sign exhibited the highest incidence of missing data, followed closely by the RB-32 sign.

Furthermore, the analysis of road sign placement revealed a notable concern regarding the location of the RB-34 sign, particularly in proximity to the onset of the taper within the passing lane. This specific location exhibited a considerable failure score, indicating a deficiency in the placement of existing signs. To be more precise, the failure score for this particular road segment reached 84%.

In specific numbers, the analysis revealed the absence of 17 RB-31 signs, 12 RB-32 signs, 5 RB-34 signs, 4 RB-37 signs, and 2 WA-33R signs. Interestingly, it was noted that there were additional signs beyond what was stipulated by the passing lane guidelines. Specifically, there were 18 additional RB-34 signs, possibly indicating the engineer’s recognition of the need for more “KEEP RIGHT EXCEPT TO PASS” signage to enhance driver awareness of the passing lane. Additionally, there were 16 extra RB-31 signs, which serve as “DO NOT PASS” warnings, alerting drivers to potentially hazardous situations when considering a passing maneuver.

A summarized overview of the results can be found in Table 6.

According to Table 6, certain error-related rules pertaining to the distance of signs from the roadside received a score below one when assessed on existing roads and before implementing the generative design process. This indicates that the placement of certain traffic signs either exceeds or falls short of the prescribed distance limit from the roadside, thus highlighting deviations from the required standards.

The analysis of the passing lanes revealed substantial enhancements in the overall score and adherence to guidelines due to implementing the generative design process and making necessary adjustments. Before employing generative design, the average initial compatibility of the considered passing lanes was 61.82%. However, this compatibility significantly increased to 91.31% after leveraging generative design techniques.

An important observation from the analysis is that all three error-related rules were fully satisfied when using the generative design approach, attaining a perfect score of 3 out of 3. This achievement highlights the effectiveness and reliability of generative design in addressing crucial safety and compliance aspects of highway infrastructure design. The generative design process effectively eliminated any violations of error-related rules, ensuring a high level of safety and adherence to standards.

While the passing lanes successfully met the error-related criteria, the analysis indicates that further optimization is required to meet warning-related rules. Despite achieving a remarkable overall score, specific warning-related aspects have room for improvement. Addressing these areas through iterative refinements in the generative design process can lead to even greater compliance with guidelines, ultimately enhancing the safety and functionality of passing lanes on highways.

The findings underscore the significance of incorporating generative design in the transportation domain. By leveraging advanced algorithms and computational methods, generative design can significantly improve infrastructure planning and design efficiency and effectiveness. It offers a powerful tool to design engineers, enabling them to create innovative and compliant solutions for highway projects.

The success of generative design in enhancing the passing lanes’ designs demonstrates its potential to revolutionize highway infrastructure planning, ensuring better compliance with guidelines and promoting safer and more efficient roadways. As the technology continues to advance, its integration into transportation projects is expected to become more prevalent, leading to smarter and more sustainable highway systems. Embracing generative design in the transportation sector has the potential to shape the future of highway infrastructure, fostering safer, more efficient, and environmentally conscious road networks.

## 6. Conclusions and Recommendations

Highway elements’ improper design is a significant contributor to traffic collisions. To tackle this issue, engineers rely on guidelines and regulations as valuable references to create compliant and safe highway models. Conventional manual methods for highway design are found to be inadequate for modern transportation networks, necessitating the exploration of alternative solutions.

The adoption of generative design, harnessing algorithms and computational methods, emerges as a promising approach to address the challenges in highway design. It enables engineers to analyze numerous scenarios and identify efficient and cost-effective solutions. Integrating Building Information Modeling (BIM) further facilitates the creation of comprehensive and precise highway models.

The specific focus of the research was on applying generative design techniques to evaluate 16 existing passing lane designs situated on Highway 3 and Highway 9 in Alberta. A series of experiments were conducted on real-world passing lane scenarios to assess the efficacy and accuracy of the generative design methodology. The initial step involved extracting the features and characteristics of the passing lanes from the available LiDAR data. Subsequently, the evaluation process commenced, which included model-checking techniques applied to the existing passing lanes. The purpose was to assess their compatibility with established guidelines. Generative design was applied to improve the passing lane designs’ compliance with the guidelines. This involved using algorithms and computational methods to generate alternative designs that adhere to the recommended guidelines. By implementing generative design, the research aimed to determine its efficacy in producing passing lane designs that meet the established criteria.

Detailed information was provided for the passing lane scenario, including highway location, start and end coordinates, passing lane length, initial real-world score, score after generative design implementation, the runtime of the generative design process, and the number of checks performed. Visual representations were also presented, such as the passing lane’s location, raw point-cloud data, filtered point-cloud, and the generative design-generated model.

The findings demonstrated substantial improvements in compliance with design guidelines after employing generative design. The implementation of generative design led to a remarkable increase in compatibility, elevating the initial 61.82% to an impressive 91.31%. This success validated the effectiveness of the generative design approach. While the generative design method effectively addressed error-related criteria, areas for further optimization were identified concerning warning-related rules.

### 6.1. Research Contributions

An innovative approach has been developed, utilizing automated techniques to evaluate designs and provide recommendations for improved compliance, leveraging LiDAR data. This methodology has been demonstrated through the presentation of 16 illustrative examples as a proof of concept. These examples serve to showcase the capability of enhancing design compliance using the proposed method, which is facilitated by a logic-based computer tool known as the BIM-kit. Notably, the application of the BIM-kit in the transportation sector represents a novel approach, underscoring the effectiveness of this innovative methodology.

In conclusion, this paper emphasizes the significance of integrating generative design into highway design and operation, offering engineers the means to create innovative and compliant solutions for highway development. By harnessing advanced algorithms, generative design substantially improves the efficiency and effectiveness of infrastructure design, ultimately contributing to the establishment of safer and more dependable transportation systems. This concept can also be applied to other types of transportation networks.

### 6.2. Research Limitations and Future Recommendations

No study is without its constraints, and by openly recognizing these limitations, we ensure transparency and a complete understanding of the study’s scope and potential implications. One limitation is the current availability of limited information, primarily comprising *XYZ* coordinates and manual grouping based on file organization. Although manual grouping allows for the basic separation of signs from the road, accurately labelling each road segment remains a challenge. Despite attempts to use clustering techniques to differentiate signs from the road, determining the exact road label remains uncertain. The lack of precise labelling for individual points poses a significant obstacle in accurately determining the exact nature of the road, especially when dealing with point clouds containing missing data, leading to irregular and unpredictable shapes. The ideal scenario would involve precise labelling with essential attributes, such as *XYZ* coordinates, directional information, and explicit indications of their belongingness to specific elements, such as road points or lines. However, the current state of available data falls short of this desirable standard. To address this challenge, one viable approach involves the investigation of semantic segmentation techniques, which have the capability to discern and categorize distinct objects within the LiDAR point cloud. Such an approach offers the potential for a finer level of data comprehension. Another avenue for resolution is the utilization of machine learning and deep learning methodologies. These algorithms can be subjected to training processes aimed at discerning patterns and characteristics within the LiDAR dataset, thereby enhancing the precision of road segment labeling.

Another limitation is the potential for improvement in the analysis using the closest point, rather than the center, for the LeftSideOf and RightSideOf methods. This adjustment could enhance the accuracy of the analysis by considering the specific point closest to the object’s center. Although this refinement has not been implemented yet, it has minimal impact on the system’s functionality.

Another significant limitation lies in the critical challenge of precise interpretation of terms and definitions utilized within relation functions, as this profoundly impacts the importance of compliance checking. The effectiveness of these relation functions is intricately linked to the clarity and precision of the language employed in the rules. Ensuring unambiguous rule sets with minimal room for misinterpretation becomes imperative during compliance checks. Thus, establishing clear and standardized definitions for the terms used in the rules becomes essential in addressing this issue. To address this challenge, it is important to acknowledge that language and terminology may undergo changes over time. Consequently, it is advisable to conduct periodic assessments and revisions of the definitions and language incorporated into relation functions, ensuring their alignment with current industry standards and emerging best practices. Additionally, a viable approach involves the integration of quality assurance checks during the rule creation process to guarantee that the language and terminology employed in relation functions adhere to predefined benchmarks for clarity and precision.

Another important aspect to consider pertains to the divergence of traffic sign placements from established guidelines. It is reasonable to assume that the engineer responsible for these decisions had specific, well-founded reasons for deviating from the prescribed guidelines. It is essential to underscore that these guidelines, while informative, are not legally mandated codes. Adhering to the guidelines does not necessarily equate to the optimal design for the given circumstances. For instance, constraints such as land ownership on either side of the road may pose challenges in ensuring that shoulder widths align precisely with the guidelines. Additionally, topographical features, like cliffs, hills, and other natural landscape elements, can introduce complexities that influence the design of passing lanes. Furthermore, design modifications may be driven by the objective of enhancing road resilience or sustainability, further emphasizing the multifaceted considerations that engineers must weigh when making these decisions.

Another noteworthy challenge encountered in this study pertains to the necessity of converting point clouds into meshes to facilitate compatibility with the BIM-kit. Given the extensive point density associated with roads, especially considering their considerable length, utilizing bounding shapes alone would result in a single, encompassing volume, which is not conducive for precise representation. Consequently, a strategic approach was adopted to segment road sections into subsegments, subsequently defining relation functions based on this segmentation. It is worth noting that, for future endeavors, an exploration of more streamlined methods and the formulation of additional relation functions may obviate the need for such extensive road segmentation, offering a more efficient and user-friendly solution.

In the context of future research endeavors, it is highly advisable to explore diverse segments of the transportation network while adhering to the pertinent guidelines specific to each respective area. This approach would yield valuable insights and serve to validate the adaptability of the generative design methodology across various contexts. Furthermore, enhancing the research by integrating more extensive and precise data sources for the precise labeling of points would bolster the robustness of findings and broaden the potential applications of the generative design approach.

Moreover, it is recommended to investigate methods for the direct importation of point cloud data into the BIM-kit without the necessity of intermediate conversion to mesh structures and partitioning. This streamlined approach could potentially offer significant advantages and efficiencies in the utilization of point cloud data within the context of Building Information Modeling (BIM) processes.

## Figures and Tables

**Table 1:** Traffic Signs.

Traffic Sign Name	Description	Shape
RB-34	KEEP RIGHT EXCEPT TO PASS	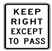
RB-37	PASSING LANE AHEAD	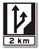
WA-33R	RIGHT LANE ENDS	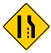
RB-31	DO NOT PASS	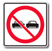
RB-32	PASSING PERMITTED	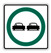

**Table 2:** Geometrical Relations for Rule Expressiveness.

Relation	Description
RoadPathDistance	Calculates distance along the road network from start to goal points.
LeftSideOf	Evaluates if a point is on the left side of an object based on vector cross product.
RightSideOf	Determines if a point is on the right side of an object.
IsAbove	Determines if one object is positioned above another.

**Table 3:** Object Description.

Object	Description
RB-37 (rb37)	Sign (PASSING LANE AHEAD)
RB-34 (rb34)	Sign (KEEP RIGHT EXCEPT TO PASS)
RB-32 (rb32)	Sign (PASSING PERMITTED)
RB-31 (rb31)	Sign (DO NOT PASS)
WA-33R (wa33r)	Sign (RIGHT LANE ENDS)
Road (road)	Marking
RoadDivergePointStart (rdps)	Virtual (Beginning of Divergence of Passing Lane)
RoadDivergePointEnd (rdpe)	Virtual (End of Divergence of Passing Lane)
RoadConvergePointStart (rcps)	Virtual (Beginning of Convergence of Passing Lane)
RoadConvergePointEnd (rcpe)	Virtual (End of Convergence of Passing Lane)

**Table 4:** Rules.

Rule Name	Description	Logical Expression
RB-34 Location 1	All RoadDivergePointStart (Passing Lane Taper), Any RB-34	ALL rdps = RoadDivergePointStart ANY rb34 = Sign with {Name CONTAINS RB-34} (rdps and rb34 MUST_HAVE FacingAngleTo EQUAL 90DEG)
RB-34 Location 2	All RoadDivergePointEnd, Any RB-34	ALL rdpe = RoadDivergePointEnd ANY rb34 = Sign with {Name CONTAINS RB-34} (rdpe and rb34 MUST_HAVE FacingAngleTo EQUAL 90DEG)
RB-34 roadside	All RB-34, Any Road, RightOf = True	ALL rb34 = Sign with {Name CONTAINS RB-34} ANY road = Road (rb34 and road MUST_HAVE RightSideOfXY EQUAL True AND rb34 and road MUST_HAVE Distance LESS_THAN_OR_EQUAL 3.5M AND rb34 and road MUST_HAVE Distance GREATER_THAN_OR_EQUAL 1.8M)
RB-37 Location	All RoadDivergePointStart, Any RB-37, Distance = 2 km	ALL rdps = RoadDivergePointStart ANY rb37 = Sign with {Name CONTAINS RB-37} (rdps and rb37 MUST_HAVE RoadPathDistance EQUAL 2000M)
RB-37 roadside	All RB-37, Any Road, RightOf = True	ALL rb37 = Sign with {Name CONTAINS RB-37} ANY road = Road (rb37 and road MUST_HAVE RightSideOfXY EQUAL True AND rb37 and road MUST_HAVE Distance LESS_THAN_OR_EQUAL 3.5M AND rb37 and road MUST_HAVE Distance GREATER_THAN_OR_EQUAL 1.8M)
RB-31 Location 1	All RoadDivergePointEnd, Any RB-31	ALL rdpe = RoadDivergePointEnd ANY rb31 = Sign with {Name CONTAINS RB-31} (rdpe and rb31 MUST_HAVE FacingAngleTo EQUAL 90DEG)
RB-31 Location 2	All RoadConvergePointStart, Any RB-31, Distance <= 120 m	ALL rcps = RoadConvergePointStart ANY rb31 = Sign with {Name CONTAINS RB-31} (rcps and rb31 MUST_HAVE RoadPathDistance LESS_THAN_OR_EQUAL 120M)
RB-31 Location 3	All RoadConvergePointEnd, Any RB-31	ALL rcpe = RoadConvergePointEnd ANY rb31 = Sign with {Name CONTAINS RB-31} (rcpe and rb31 MUST_HAVE FacingAngleTo EQUAL 90DEG)
RB-31 roadside	All RB-31, Any Road, LeftOf = True	ALL rb31 = Sign with {Name CONTAINS RB-31} ANY road = Road (rb31 and road MUST_HAVE LeftSideOfXY EQUAL True AND rb31 and road MUST_HAVE Distance LESS_THAN_OR_EQUAL 3.5M AND rb31 and road MUST_HAVE Distance GREATER_THAN_OR_EQUAL 1.8M)
RB-32 Location	All RoadDivergePointStart (Shoulder Taper), Any RB-32	ALL rdps = RoadDivergePointStart ANY rb32 = Sign with {Name CONTAINS RB-32} (rdps and rb32 MUST_HAVE FacingAngleTo EQUAL 90DEG)
RB-32 roadside	All RB-32, Any Road, LeftOf = True	ALL rb32 = Sign with {Name CONTAINS RB-32} ANY road = Road (rb32 and road MUST_HAVE LeftSideOfXY EQUAL True AND rb32 and road MUST_HAVE Distance LESS_THAN_OR_EQUAL 3.5M AND rb32 and road MUST_HAVE Distance GREATER_THAN_OR_EQUAL 1.8M)
Road Split Length	The length of the split road segment should be between 1.5 and 2 km	ALL rdps = RoadDivergePointStart ALL rcpe = RoadConvergePointEnd (rdps and rcpe MUST_HAVE RoadPathDistance GREATER_THAN_OR_EQUAL 1500M AND rdps and rcpe MUST_HAVE RoadPathDistance LESS_THAN_OR_EQUAL 2000M)
Sign Road Dist	Sign distance to roadside	ALL sign = Sign ANY road = Road (sign and road MUST_HAVE Distance LESS_THAN_OR_EQUAL 3.5M AND sign and road MUST_HAVE Distance GREATER_THAN_OR_EQUAL 1.8M)
Sign Road Rule	All Sign, All Road, IsAbove = False	ALL sign = Sign ALL road = Road (sign and road MUST_HAVE IsAbove EQUAL False)
Sign to Sign Distance	Equal signs	ALL s1 = Sign ALL s2 = Sign (s1 and s2 MUST_HAVE Distance GREATER_THAN 1M)
WA-33R Location 1	All RoadConvergePointStart, Any WA-33R, Distance = 300 m	ALL rcps = RoadConvergePointStart ANY wa33r = Sign with {Name CONTAINS WA-33R} (rcps and wa33r MUST_HAVE RoadPathDistance EQUAL 300M)
WA-33R Location 2	All RoadConvergePointStart, Any WA-33R	ALL rcps = RoadConvergePointStart ANY wa33r = Sign with {Name CONTAINS WA-33R} (rcps and wa33r MUST_HAVE FacingAngleTo EQUAL 90DEG)
WA-33R roadside	All WA-33R, Any Road, RightOf = True	ALL wa33r = Sign with {Name CONTAINS WA-33R} ANY road = Road (wa33r and road MUST_HAVE RightSideOfXY EQUAL True AND wa33r and road MUST_HAVE Distance LESS_THAN_OR_EQUAL 3.5M AND wa33r and road MUST_HAVE Distance GREATER_THAN_OR_EQUAL 1.8M)

**Table 5:** Passing lane rule evaluation scores.

Rule Name	Scores		Rule Name	Score	
	Before	After		Before	After
Sign Road Dist	0.61 (Failed)	1.00 (Passed)	RB-31 Location 2	1.00 (Passed)	1.00 (Passed)
Sign Road Rule	1.00 (Passed)	1.00 (Passed)	RB-31 Location 3	0.40 (Failed)	1.00 (Passed)
Sign to Sign Distance	1.00 (Passed)	1.00 (Passed)	RB-31 roadside	0.67 (Failed)	1.00 (Passed)
RB-34 Location 1	0.11 (Failed)	0.99 (Failed)	RB-32 Location	0.11 (Failed)	1.00 (Passed)
RB-34 Location 2	0.40 (Failed)	0.39 (Failed)	RB-32 roadside	0.67 (Failed)	1.00 (Passed)
RB-34 roadside	0.41 (Failed)	1.00 (Passed)	Road Split Length	1.00 (Passed)	1.00 (Passed)
RB-37 Location	0.99 (Failed)	1.00 (Passed)	WA-33R Location 1	0.66 (Failed)	1.00 (Passed)
RB-37 roadside	0.68 (Failed)	1.00 (Passed)	WA-33R Location 2	0.40 (Failed)	0.39 (Failed)
RB-31 Location 1	0.03 (Failed)	1.00 (Passed)	WA-33R roadside	0.41 (Failed)	1.00 (Passed)

**Table 6:** Modelling Results.

Passing Lane Test #	Initial Score	Score after Generative Design
1	Total Score: 7.27/12: 60.58% Error: 2.60/3.00 Warning: 4.67/9.00	Total Score: 16.77/18: 93.15% Errors: 3.00/3.00 Warning: 13.77/15.00
2	Total Score: 7.04/12: 58.70% Error: 2.60/3.00 Warning: 4.45/9.00	Total Score: 16.96/18: 94.20% Errors: 3.00/3.00 Warning: 13.96/15.00
3	Total Score: 8.12/12: 67.64% Error: 2.59/3.00 Warning: 5.52/9.00	Total Score: 17.18/18: 95.44% Errors: 3.00/3.00 Warning: 14.18/15.00
4	Total Score: 8.01/12: 66.73% Error: 2.60/3.00 Warning: 5.41/9.00	Total Score: 17.20/18: 95.58% Errors: 3.00/3.00 Warning: 14.20/15.00
5	Total Score: 9.52/16: 59.48% Error: 2.60/3.00 Warning: 6.91/13.00	Total Score: 16.28/18: 90.47% Errors: 3.00/3.00 Warning: 13.28/15.00
6	Total Score: 9.12/16: 57.00% Error: 2.59/3.00 Warning: 6.53/13.00	Total Score: 14.76/18: 81.99% Errors: 3.00/3.00 Warning: 11.76/15.00
7	Total Score: 10.55/18: 58.61% Error: 2.62/3.00 Warning: 7.94/15.00	Total Score: 16.78/18: 93.20% Errors: 3.00/3.00 Warning: 13.78/15.00
8	Total Score: 9.72/14: 69.46% Error: 2.64/3.00 Warning: 7.08/11.00	Total Score: 17.11/18: 95.03% Errors: 3.00/3.00 Warning: 14.11/15.00
9	Total Score: 10.11/16: 63.16% Error: 2.72/3.00 Warning: 7.39/13.00	Total Score: 15.73/18: 87.37% Errors: 3.00/3.00 Warning: 12.73/15.00
10	Total Score: 8.22/18: 45.67% Error: 2.63/3.00 Warning: 5.59/15.00	Total Score: 15.59/18: 86.60% Errors: 3.00/3.00 Warning: 12.59/15.00
11	Total Score: 10.56/16: 66.01% Error: 2.63/3.00 Warning: 7.93/13.00	Total Score: 17.18/18: 95.47% Errors: 3.00/3.00 Warning: 14.18/15.00
12	Total Score: 10.57/18: 58.70% Error: 2.63/3.00 Warning: 7.94/15.00	Total Score: 16.30/18: 90.55% Errors: 3.00/3.00 Warning: 13.30/15.00
13	Total Score: 10.71/16: 66.93% Error: 2.68/3.00 Warning: 8.03/13.00	Total Score: 16.45/18: 91.41% Errors: 3.00/3.00 Warning: 13.45/15.00
14	Total Score: 9.07/14: 64.77% Error: 2.66/3.00 Warning: 6.410/11.00	Total Score: 16.93/18: 94.07% Errors: 3.00/3.00 Warning: 13.93/15.00
15	Total Score: 10.08/16: 63.00% Error: 2.62/3.00 Warning: 7.46/13.00	Total Score: 15.58/18: 86.54% Errors: 3.00/3.00 Warning: 12.58/15.00
16	Total Score: 8.78/14: 62.68% Error: 2.64/3.00 Warning: 6.14/11.00	Total Score: 16.17/18: 89.83% Errors: 3.00/3.00 Warning: 13.17/15.00

## Data Availability

The authors confirm that the data supporting the conclusions of this study are available upon request from Alberta Transportation.
